# Emotion-Aware Virtual Reality Through Multimodal ECG and Postural Fusion

**DOI:** 10.3390/s26185726

**Published:** 2026-09-09

**Authors:** Juan Benavides, Mayra Carrión-Toro, Cindy López, David Morales-Martínez, Marco Santórum, Patricia Acosta-Vargas

**Affiliations:** 1 Departamento de Informática y Ciencias de la Computación, Escuela Politécnica Nacional, Quito 170525, Ecuador; juan.benavides@epn.edu.ec (J.B.); mayra.carrion@epn.edu.ec (M.C.-T.); cindy.lopez@epn.edu.ec (C.L.); david.morales03@epn.edu.ec (D.M.-M.); marco.santorum@epn.edu.ec (M.S.); 2 Intelligent and Interactive Systems Laboratory, Universidad de Las Américas, Quito 170503, Ecuador

**Keywords:** affective computing, virtual reality, emotion regulation, digital mental health, multimodal emotion recognition, electrocardiogram (ECG), postural analysis, SDG 3

## Abstract

Immersive Virtual Reality (VR) environments are increasingly adopted in clinical psychology and stress-management contexts; however, their therapeutic effectiveness depends on the system’s ability to understand and dynamically respond to users’ affective states. Emotional regulation plays a key role in psychological resilience, directly influencing stress coping mechanisms, cognitive performance, and overall mental well-being. Despite recent advances, automatic recognition of scenario-associated affective conditions in VR remains challenging because head-mounted displays occlude facial features. This study proposes a VR-based serious game for affective training and regulation, in which users interact with goal-oriented scenarios targeting fear, anger, and joy. We introduce a multimodal affective computing model to objectively assess users’ emotional responses by integrating electrocardiogram (ECG) signals and posture-based features extracted through computer vision. An early-fusion architecture combined with a Long Short-Term Memory (LSTM) network captures temporal dependencies in synchronized multimodal data. We established a controlled experimental framework using immersive VR scenarios, enabling the collection of synchronized physiological and behavioral data from a cohort of 20 healthy adult participants. The proposed model was evaluated under a strict Leave-One-Subject-Out (LOSO) cross-validation scheme across independent subjects, achieving a robust inter-subject accuracy of 78.94%±10.77% and a global macro F1-score of 0.635, demonstrating strong generalization to entirely unseen users without data leakage. Furthermore, the system maintained an outstanding balance in detecting active emotional states (recall > 80.0% for fear, anger, and joy). Additionally, subjective evaluations using the PANAS and SGU questionnaires confirmed the coherence between detected and perceived emotional states, as well as the system’s high usability. The results suggest the potential viability of combining immersive environments and multimodal affective computing to explore the technical feasibility of adaptive frameworks that could eventually translate into healthcare contexts. This work may contribute to the development of intelligent digital health technologies by providing a foundation for responsive VR systems that can monitor emotional regulation and are fully aligned with sustainable well-being ecosystems (SDG 3: Good Health and Well-being and SDG 10: Reduced Inequalities).

## 1. Introduction

The convergence of multimodal affective computing, immersive Virtual Reality (VR), and deep learning architectures is actively transforming the design of exploratory frameworks for mental health monitoring and emotion-regulation therapies [1]. Virtual environments are no longer viewed merely as visual displays, but as advanced affective platforms capable of evoking profound emotional involvement through heightened psychological presence [1]. Consequently, integrating affective computing into immersive systems has become a key component of developing emotion-aware and adaptive immersive environments, particularly in serious games and training applications [2,3].

Automatic emotion recognition has become an essential capability in modern information systems, allowing digital environments to adapt to the user’s affective state and enhance experiences in healthcare, education, training, and human–computer interaction. This capability is particularly relevant in immersive virtual reality environments, where emotional responses directly influence participation, performance, and perception of presence [1,4,5]. Therefore, systems that can interpret emotions in real time are fundamental to developing user-centered, adaptive training and exploratory emotional regulation platforms.

However, emotion recognition in VR remains an open challenge. Traditional approaches based on facial expressions perform poorly on head-mounted displays (HMDs), which occlude most of the face and hinder the extraction of affective visual cues, thereby reducing accuracy to near 60% [6]. In addition, unimodal approaches based solely on physiological or visual signals are highly sensitive to noise, motion artifacts, and inter-subject variability, limiting their robustness in real-world scenarios [7,8]. Although multimodal approaches have gained increasing attention, most existing solutions are not specifically designed for immersive VR under facial occlusion and rarely incorporate posture-related information, despite its relevance in the motor expression of emotions.

In parallel, VR-based serious games have emerged as promising tools for emotional engagement and training, enabling users to interact with controlled scenarios that can elicit and potentially regulate affective states such as fear, anger, and joy [2]. Nevertheless, a key limitation of these systems is the lack of objective mechanisms to validate whether intended emotional states are effectively elicited during user interaction. This gap limits the development of intelligent, closed-loop applications that dynamically adapt stimuli in response to real-time emotional biofeedback.

To address these challenges, this work proposes a multimodal Deep Learning model that classifies scenario-associated affective conditions in VR users by integrating two complementary channels: electrocardiogram (ECG) signals and posture-based features extracted via computer vision. The proposed architecture uses an early-fusion strategy, combining physiological and body-movement representations into a unified multimodal sequence, then processing it with a Long Short-Term Memory (LSTM) network to capture temporal dependencies across modalities.

This study is a pilot feasibility validation designed to evaluate the technical viability of the proposed sensor and multimodal pipeline. This exploratory evaluation was conducted with a cohort of 20 healthy young adults, which introduces specific boundary conditions that prevent direct generalization to broader clinical populations. First, the recruitment pool was restricted to healthy participants, meaning autonomic and motor responses may vary in clinical or pathological cohorts. Second, our sample presents age restrictions (20–32 years) and potential recruitment bias common in university environments. Finally, individual factors such as prior familiarity with VR environments (35% of our cohort) and physical habituation were not controlled as active experimental variables. Consequently, this work does not aim to establish a universally generalized diagnostic system, but rather to prove the technical feasibility and accuracy of combining low-cost ECG telemetry and skeletal tracking under strict user-independent conditions (Leave-One-Subject-Out) as a foundation for future, larger-scale clinical trials.

The model is validated in a VR-based serious game environment developed in Unity for the Meta Quest 3 headset, where immersive, goal-oriented scenarios are designed to elicit and engage emotional states such as fear, anger, and joy. The central hypothesis is that combining ECG and posture information improves affective classification performance compared to unimodal approaches, particularly in contexts where facial expressions are unavailable.

Beyond technical performance, this work contributes to broader technological goals such as Good Health and Well-being (SDG 3), specifically targeting digital mental health intervention frameworks, alongside Reduced Inequalities (SDG 10) through non-invasive, low-cost sensing.

## 2. Background and Related Work

Affective computing in immersive Virtual Reality (VR) environments requires robust models that can interpret emotional states without relying on facial features, given the occlusion caused by head-mounted displays (HMDs). To position this work, this section reviews multimodal affective computing approaches, physiological and behavioral signals, and recent developments in emotion recognition in VR environments.

### 2.1. Multimodal Affective Computing

Multimodal models integrate heterogeneous data sources, such as physiological signals, behavioral patterns, and visual information, to improve the robustness and accuracy of emotion recognition systems [9,10]. These approaches have outperformed unimodal models by capturing complementary information across modalities.

Fusion strategies are commonly categorized as early, intermediate, and late fusion. Early fusion combines features at the input level, intermediate fusion integrates modality-specific representations at hidden layers, and late fusion aggregates the outputs of independent classifiers [11,12]. Despite their effectiveness, these strategies remain limited in immersive environments, particularly for synchronized multimodal data under VR constraints.

### 2.2. Physiological Signals for Emotion Recognition

Physiological signals are widely used in affective computing because they capture internal emotional responses. Among them, electrocardiogram (ECG) signals provide valuable information about heart rate variability and autonomic nervous system activity, which are closely related to emotional states such as stress, fear, and relaxation [7,13].

Previous studies indicate that ECG-based emotion recognition systems can achieve accuracies between 70% and 85%, depending on the experimental conditions and classification models [14]. However, most of these studies are conducted in controlled or non-immersive environments, limiting their applicability to real-time VR scenarios.

### 2.3. Postural and Behavioral Signals

In addition to physiological signals, body posture and movement patterns have emerged as relevant indicators of emotional expression. Emotions such as fear, tension, and joy are often reflected through body dynamics and spatial behavior [15].

Recent works demonstrate that posture-based approaches can achieve high classification accuracy when applied independently. For example, Wu et al. [16] reported accuracies of up to 90% using upper-body skeletal representations. Despite this potential, limited research integrates postural features with physiological signals such as ECG, particularly in immersive VR environments.

### 2.4. Virtual Reality and Emotion Recognition

Virtual Reality enables the creation of immersive environments that intensify emotional responses by combining sensory stimuli and interactive elements [17,18]. Its application has expanded to domains such as healthcare, training, and education, where emotional engagement plays a key role.

However, emotion recognition in VR presents significant challenges. Occlusion caused by head-mounted displays reduces the reliability of face-based approaches, necessitating alternative sensing modalities, such as physiological and behavioral signals. Additionally, the lack of standardized datasets and real-time multimodal frameworks hinders the development of adaptive, emotion-aware systems in immersive environments.

Recent studies exploring emotion recognition in VR report moderate performance, typically between 65% and 75%, highlighting the difficulty of generalizing models in dynamic and interactive scenarios [19,20,21].

### 2.5. Deep Learning for Multimodal Emotion Recognition

Deep learning models, particularly recurrent neural networks such as Long Short-Term Memory (LSTM), are widely used for modeling temporal dependencies in physiological and behavioral data [22,23]. These models are especially effective in capturing sequential patterns and have been successfully applied in multimodal emotion recognition tasks.

However, few studies have explored LSTM-based architectures trained on synchronized multimodal data collected in immersive VR environments, highlighting the need for models that integrate temporal and cross-modal information under realistic conditions.

### 2.6. Research Gaps and Novelty Motivation

Despite recent advances in multimodal affective computing, a comprehensive review of the literature reveals critical technical and methodological gaps that limit current frameworks [24]. The core novelty of this work stems directly from overcoming the fundamental trade-offs among tracking accuracy, hardware invasiveness, and facial-occlusion immunity that characterize existing solutions [24].

As synthesized in Table 1, conventional emotion recognition relies heavily on paradigms that fail under real-world VR immersion. While facial-expression models (used in 9 core contemporary studies) provide a direct communication channel, they suffer from a drastic drop in accuracy (falling below 60%) due to physical occlusion by head-mounted displays (HMDs) that cover the upper face [19]. Concurrently, neural-centric architectures (dominant in 18 major frameworks) utilize complex multi-channel EEG caps which, despite their robustness, introduce extreme user friction, high equipment costs, and severe sensitivity to motion artifacts during interactive, task-driven scenarios [20,21].

Our proposed framework introduces a paradigm shift by completely bypassing facial metrics and complex neural setups. Instead, it pioneers the synchronization of non-invasive cardiac (ECG) with markerless behavioral body gestures (2D posture tracking via MediaPipe) captured through an external camera [24,25]. While upper-body tracking has shown isolated success in improving safety and ergonomics [16], its cross-modal fusion with raw ECG sequences to address VR facial occlusion remains underexplored [24]. Furthermore, while most related works validate their models on static, passive datasets, achieving standard cross-subject accuracies between 65% and 75% [26,27], our attention-based bidirectional LSTM network achieves a robust 78.94% accuracy under a strict Leave-One-Subject-Out (LOSO) cross-validation scheme within an active, task-driven serious game environment [24].

**Table 1:** Comparative analysis of operational constraints, sensing modalities, and validation schemes across affective computing paradigms in VR.

Paradigm	Facial Occlusion	Sensing Setup	Fusion	VR Stimulus	Accuracy
Facial-Expression Models [19]	High (HMD occlusion)	Optical Action Units (AUs)	No	Passive/ Interactive	<60%
Neural Frameworks (EEG) [20,21]	None	Multi-channel EEG	Rare	Passive/Static	65–75%
Passive Cardiac Fusions [28,29]	None	Single-channel ECG/PPG	No	Passive	70–85%
Proposed Framework (This Work)	None	Single-channel ECG + 2D Posture	Yes	Active Serious Game	78.94%

## 3. Proposed Multimodal Emotion Detection Model

Recent advances in affective computing have shown the potential of individual modalities, such as physiological signals and behavioral cues, for emotion recognition. However, most existing approaches rely on unimodal data or controlled experimental conditions, limiting their applicability in immersive Virtual Reality (VR) environments where facial information is unavailable.

In parallel, VR-based serious games have emerged as promising tools for emotional engagement and training, enabling users to interact with controlled scenarios that elicit specific affective states. Nevertheless, these systems lack objective mechanisms to validate whether they effectively induce the intended emotions during user interaction.

To address these limitations, this work proposes a multimodal Deep Learning model that integrates electrocardiogram (ECG) signals with posture-based features to enable robust emotion recognition in immersive VR environments under facial occlusion.

### 3.1. VR Stimulus—Game

This work proposes a multimodal model that combines ECG signals and postural analysis to classify users’ emotions in virtual reality applications or games. In this context, the VR stimulus plays a fundamental role by enabling the induction and recording of the emotional responses required for classification.

**Figure 1:**
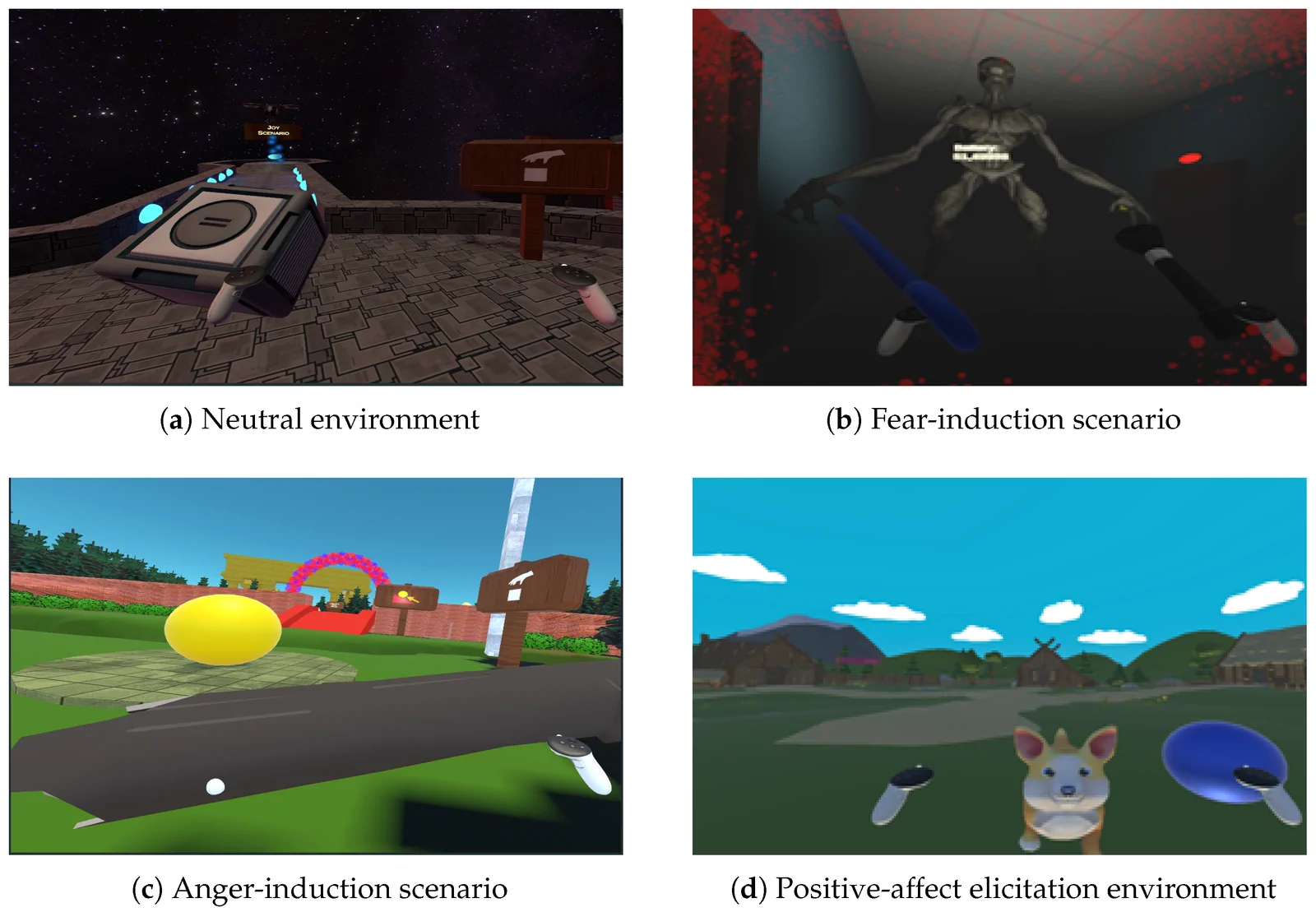
Virtual reality scenarios designed for emotional elicitation: (**a**) Neutral environment, (**b**) fear-induction scenario, (**c**) anger-induction scenario, and (**d**) positive-affect elicitation environment.

To this end, we developed four virtual reality scenarios for the Meta Quest 3 headset, each designed to evoke a specific emotional state through controlled interaction mechanics and immersive environmental conditions. Figure 1 illustrates the VR environments used for emotional elicitation, including: (a) a neutral environment, (b) a fear-induction scenario, (c) an anger-induction scenario, and (d) a positive-affect elicitation environment.

The neutral environment shown in Figure 1a was designed as an exploratory space station with low visual intensity and ambient electronic background audio. This environment allowed participants to become familiar with locomotion, object interactions, and Meta Quest 3 controls while reducing novelty effects before full exposure. Additionally, users could explore the environment and select the scenario to be played.

The fear-induction scenario presented in Figure 1b was designed around stress, uncertainty, and survival-oriented interaction mechanics. Participants were required to escape from a dark, confined environment while avoiding a hostile creature that could trigger jump scares. To progress, users needed to search for hidden keys to unlock doors while managing limited flashlight battery resources. Players could collect additional batteries throughout the environment and use specific interactive objects to temporarily distract the enemy. Failure led to a complete restart of the scenario, thereby reinforcing psychological tension and emotional pressure throughout the experience.

The anger-induction scenario illustrated in Figure 1c focused on eliciting frustration, a sense of challenge, and a perceived loss of control during user interaction. Participants were tasked with guiding a ball through a multi-stage environment without direct manipulation; instead, they interacted indirectly via virtual tools, such as sticks or elongated objects, to push the ball toward a designated target area. To introduce systematic cognitive friction, certain interaction tools intentionally exhibited inconsistent physics-based behaviors, forcing participants to adapt dynamically through trial and error. The environment combined wide sections, where users were required to construct bridges or manipulate physics-driven structures, with narrow, highly constrained pathways where losing control of the ball forced it to return to the previous checkpoint. Consequently, these design mechanics successfully promoted sustained frustration, precision-induced stress, and emotional tension.

Finally, the positive-affect elicitation environment illustrated in Figure 1d was designed as a relaxed, low-stress interaction space centered around a virtual pet companion. Participants interacted with an animated dog-like character that responded dynamically and positively to user actions, such as retrieving a thrown ball or engaging in playful cooperative tasks. The virtual scenario incorporated high-brightness visual design, uplifting auditory stimuli, and a complete absence of punitive mechanics to systematically encourage calmness, positive engagement, and emotional comfort. Notably, due to its high-reward, low-challenge architecture, prolonged exposure occasionally induced mild boredom or emotional habituation during extended interactions.

Beyond data acquisition, these scenarios were designed as controlled emotional interactive environments and challenge dynamics. These immersive environments enabled the elicitation and validation of emotional responses while providing a foundation for future emotion-aware immersive systems and affective computing applications in VR.

### 3.2. ECG Acquisition Module and Hardware Validation

The ECG acquisition module constitutes the primary physiological sensing component of the proposed multimodal emotion recognition system, enabling the real-time recording of cardiac activity while the user actively interacts with the immersive virtual reality environment. The acquired ECG signal reflects the heart’s electrical activity and captures the essential PQRST complexes needed to analyze autonomic responses during emotional stimulation.

**Figure 2:**
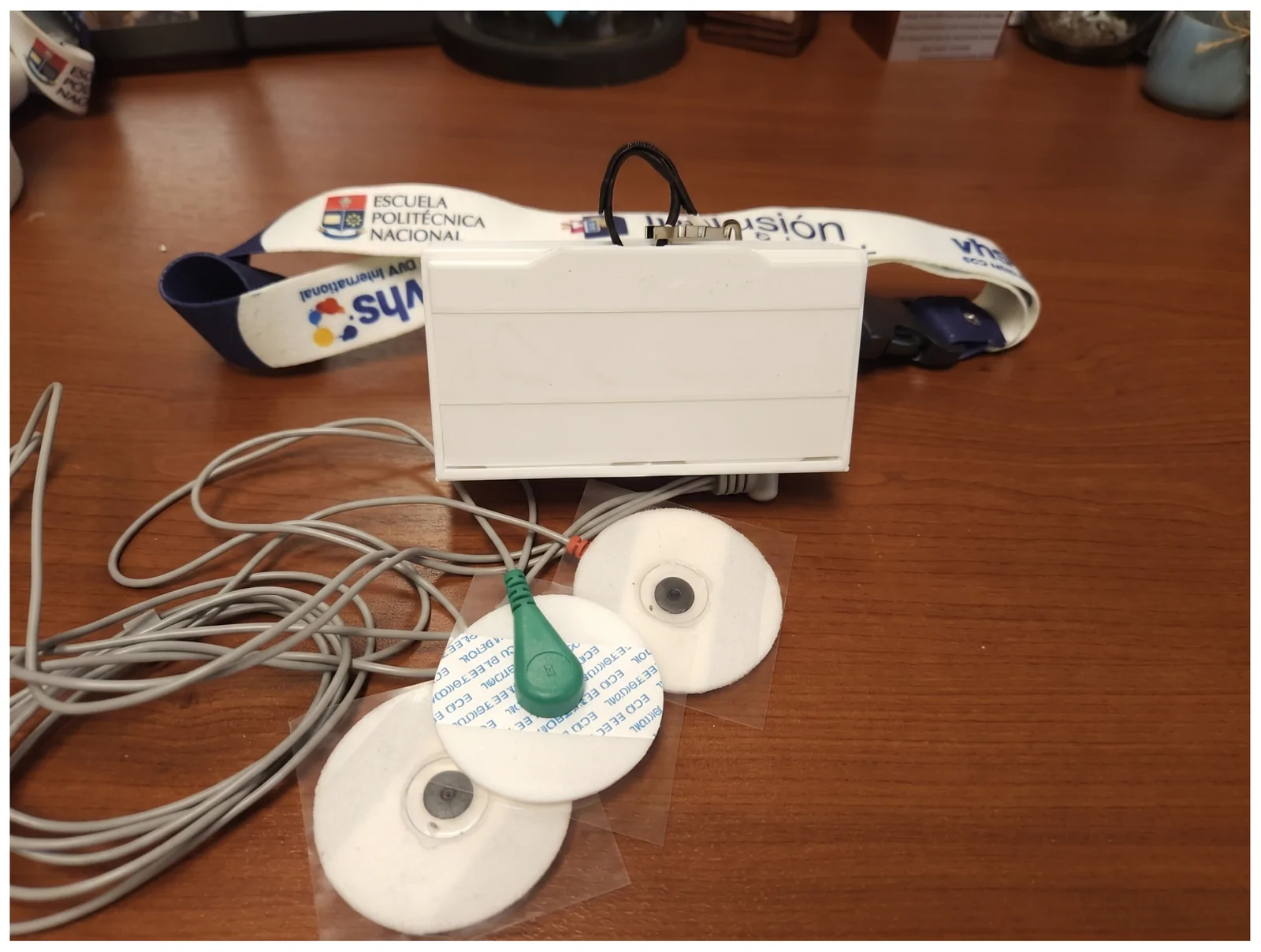
Custom ECG acquisition sensor developed for real-time physiological data collection during immersive VR interaction.

Because many commercial ECG devices are proprietary and lack open serial communication protocols for experimental synchronization, we developed a custom wireless ECG acquisition device for this study, adapted from the iPlus methodology [30]. The hardware configuration utilizes an analog front-end (AFE) AD8232 heart rate monitor integrated with a 32-bit ESP32 microcontroller. The analog signal is captured through the ESP32’s internal Analog-to-Digital Converter (ADC) at a stable sampling rate of 100 Hz (Δt=10 ms), configured with hardware-timed loops to ensure temporal sampling stability and jitter of less than ±0.5 ms. Figure 2 illustrates the resulting custom-built wearable hardware prototype.

For physical sensing, we implemented a non-invasive, three-electrode configuration to establish a Modified Einthoven Lead I setup. The two active electrodes (positive and negative) were placed on the participant’s left and right wrists, while the ground reference electrode was positioned on the upper chest area over the sternum. This specific anatomical placement represents a critical methodological and ethical design choice. Traditional chest-only electrode placements (such as Lead II) can cause significant participant discomfort, introduce placement errors when self-applied, and impose sensitive physical boundary constraints, particularly in multi-gender operational trials. By utilizing a wrist-based active setup, electrode placement was streamlined, participant burden was significantly reduced, and personal physical boundaries were strictly respected while fully satisfying the institutional ethics board guidelines (Protocol EO-083-2025). This configuration successfully maximized R-peak morphology and provided a stable cardiac telemetry stream throughout the active VR tasks.

To handle user movements during active VR interactions, the ESP32 firmware incorporates a real-time 5-point temporal moving average filter to smooth high-frequency noise and sudden baseline fluctuations prior to transmission. Additionally, the hardware-integrated Lead-Off detection circuit of the AD8232 (utilizing pins LO+ and LO-) was mapped to GPIO inputs 34 and 35. This design enables the hardware to dynamically identify electrode displacement or skin contact loss caused by physical movement, automatically setting corrupted telemetry frames to zero to preserve dataset integrity.

Data transmission is performed wirelessly via Serial Port Profile (SPP) Bluetooth Classic to a dedicated processing station at 115,200 bps. To benchmark the custom ESP32 + AD8232 sensor prototype, preliminary laboratory trials were conducted by comparing its output against a commercial research-grade benchmark platform (Shimmer3 ECG) under simultaneous, lead-matched (Modified Lead I) stationary rest conditions over 5-minute recording sessions across N=10 comparative validation participants. The Mean Absolute Error (MAE) of R-peak time displacement was computed as

(1) $$\mathrm{MAE}=\frac{1}{N_{\mathrm{beats}}}\sum_{i=1}^{N_{\mathrm{beats}}}\left|t_{\mathrm{custom},i}-t_{\mathrm{ref},i}\right|$$

yielding an MAE of 7.8ms, a baseline Signal-to-Noise Ratio (SNR) of 26.4dB, and a Pearson correlation coefficient of r=0.948. Bland–Altman agreement analysis for inter-beat intervals (RR) revealed a mean paired bias of +1.2ms with 95% limits of agreement spanning from −12.4ms to +14.8ms. These metrics confirm sufficient metrological timing precision for short-term autonomic feature extraction during interactive VR tasks without asserting full regulatory clinical equivalency.

During active VR motion, physical displacement introduces minor baseline wander and muscle artifacts, reducing the average dynamic SNR to 17.8 dB. To manage this uncertainty, the receiving software runs an active multi-threaded validation loop that restricts the telemetry stream to physiological voltage ranges ($300<V_{\mathrm{raw}}<3000$), discarding out-of-bounds samples. Multi-threaded processing manages the incoming serial stream and the 30-fps webcam video feeds in parallel, achieving a master–slave synchronization with a stable software-level temporal alignment latency of 12.4±2.1 ms, and a packet loss rate below 0.35% across all experimental sessions.

### 3.3. Postural Analysis Module

Postural analysis was performed using the MediaPipe Pose library [25], developed by Google, which enables real-time detection and tracking of human body landmarks.

Using its specialized tracking architecture, we obtained spatial coordinates for 33 joint landmarks, capturing both upper- and lower-body extremities. These coordinates were subsequently processed to evaluate geometric variations and temporal trajectories among the most relevant inertial key points, thereby identifying behavioral patterns associated with different emotional states.

This approach is particularly useful because affective states frequently manifest as subtle changes in posture and muscle tone, which can be quantified and directly correlated with physiological features extracted from the ECG stream. This behavioral modality is highly relevant in immersive VR contexts where traditional facial analysis is inaccessible due to HMD occlusion, making body movement a primary observable indicator of emotional expression.

### 3.4. Multimodal Early-Fusion LSTM Architecture

An early-fusion model based on a Long Short-Term Memory (LSTM) network was employed to simultaneously integrate features extracted from physiological signals (ECG) and postural variables. This feature-level early-fusion strategy was selected to force the network to capture direct, raw cross-modal correlations and joint feature interactions from the first processing layers, while minimizing architectural complexity to ensure low-latency performance optimized for real-time deployment [11,12].

Furthermore, this architecture enables effective capture of the complex temporal dependencies present in both data types, as cardiac activity and body movements evolve dynamically over time [11]. In parallel, the postural analysis module extracted the spatial coordinates of the upper-body joint landmarks. Using only the 2D components (X,Y) for each landmark to mitigate depth-estimation jitter, we generated a structured vector of 34 features per timestep [25]. When evaluating the feature space configuration, these 34 postural coordinates are synchronized and concatenated with 4 derived ECG time-series vectors (standardized voltage, first-order difference, absolute velocity, and instantaneous energy), yielding a unified 38-dimensional multimodal input vector per temporal instance [12]. The resulting synchronized data stream was segmented into temporal windows of 31 time steps, preserving sequential variations that constitute the primary discriminative information for emotion recognition.

The proposed deep learning model consists of a bidirectional 64-unit LSTM layer, selected for its ability to retain long-term temporal sequences while mitigating the vanishing gradient problem [22]. A Bahdanau attention mechanism was integrated over the LSTM layer to dynamically weight and highlight the most relevant emotional frames within each sequence [31]. Two subsequent fully connected dense layers with 64 and 32 neurons, respectively, utilizing ReLU activation and a 30% dropout rate, were added to reduce overfitting and improve generalization performance [32]. Finally, a softmax output layer produces the probability distribution over the four target emotions: neutral, anger, fear, and joy.

Training used the Adam optimizer, sparse categorical cross-entropy loss, and a strict cross-validation cross-subject loop to ensure stable convergence. This architecture jointly models the synchronized temporal evolution of ECG and body posture, enabling coherent multimodal classification aligned with the emotional dynamics induced in the VR scenarios. To ensure absolute reproducibility, Table 2 summarizes the execution environment, hardware baseline, and fixed hyperparameter distributions.

Experimental results demonstrate that the proposed multimodal approach achieves a robust average validation accuracy of 78.94% ± 10.77% and a global macro F1-score of 0.635 under a strict Leave-One-Subject-Out (LOSO) cross-validation evaluation scheme across 20 independent users [27], using a participant-grouped inner split to prevent overlap-induced leakage between training and validation windows. These results confirm that integrating synchronized ECG time-series and normalized postural tracking components provides a robust and reliable approach to affective computing in immersive VR environments under severe facial occlusion [19,27].

**Figure 3:**
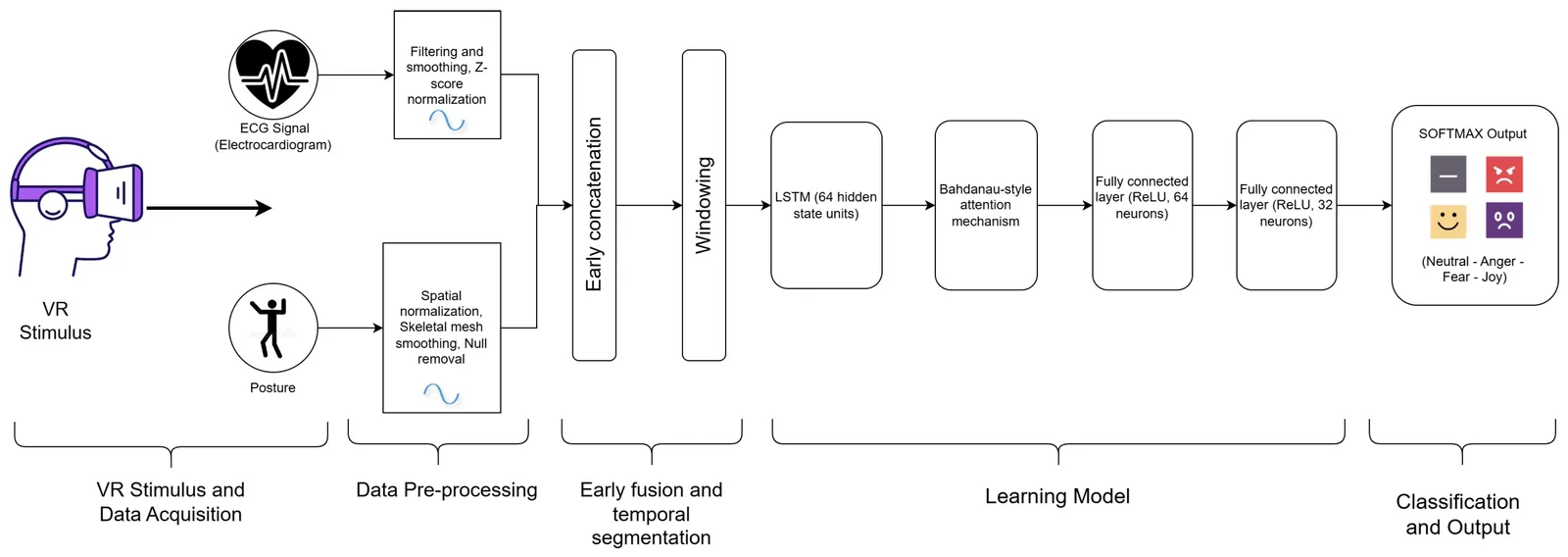
Overall architecture of the proposed early-fusion attention-based LSTM model.

Beyond classification performance, the proposed architecture provides a solid foundation for emotion-aware systems that support emotional training, clinical research, and adaptive learning ecosystems [33]. Figure 3 shows the general structure of the proposed model:

## 4. Methodology

**Figure 4:**
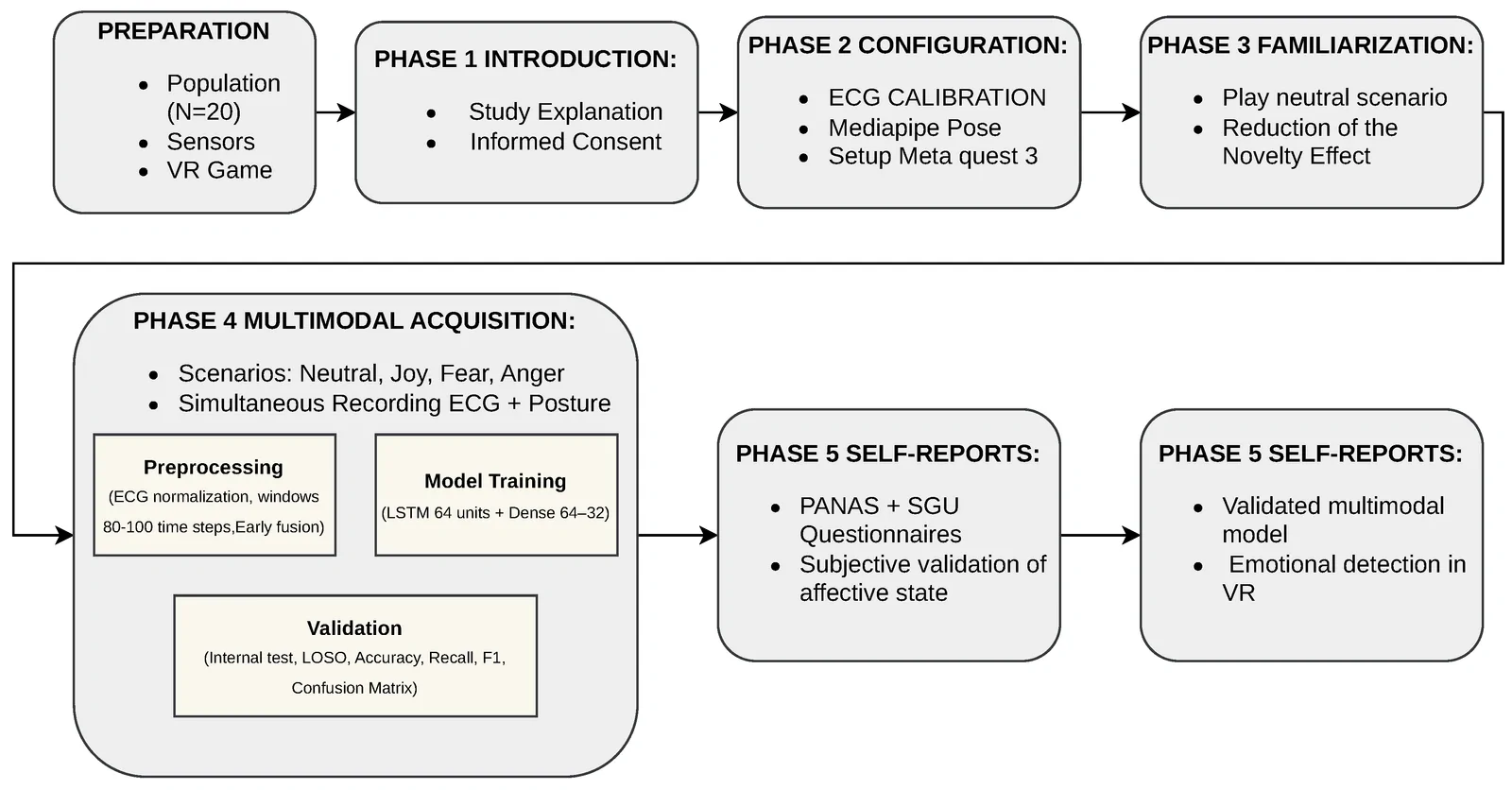
Methodology scheme.

Figure 4 summarizes the methodological workflow used in this study, comprising five interconnected phases: clinical induction preparation, multimodal setup calibration, baseline familiarization, active data acquisition, and cross-subject model validation.

### 4.1. Preparation and Participant Profile

The initially recruited experimental cohort comprised 22 adult volunteers; however, after applying strict inclusion and exclusion criteria, the final analytical sample comprised 20 non-vulnerable adult participants (N=20), with a demographic distribution of 11 males and 9 females, aged 20–32 years. In addition to screening out individuals with a self-reported history of cardiovascular or neurological disorders that could compromise autonomic or musculoskeletal responses during immersion, we enforced a technical exclusion criterion regarding optical correction. Specifically, individuals whose prescription eyeglasses could not physically fit comfortably within the Meta Quest 3 head-mounted display (HMD) chassis without inducing mechanical pressure or severe visual distortion were excluded from the trials. Within the final validated sample, 35% of the participants reported prior familiarity with operating immersive Virtual Reality (VR) environments.

A rigorous safety and voluntary withdrawal protocol was established prior to the experimental sessions. Participants were explicitly instructed that they could verbally report any symptoms of discomfort, nausea, anxiety, or motion sickness at any juncture to immediately terminate the experiment, without penalty or the need for justification. Continuous adverse-event and cybersickness monitoring was maintained through active verbal check-ins during baseline breaks between experimental scenarios.

Regarding real-time experimental anomalies, exactly two participants from the initial cohort reported acute symptoms of cybersickness and physical discomfort during the interactive tasks. In strict accordance with the safety protocol, these two individuals were immediately withdrawn from the trial; their respective testing sessions were terminated prematurely, and their recorded windowed data were discarded to preserve overall dataset integrity. The remaining 20 participants successfully completed the full interaction loop across all four immersive scenarios.

Upon concluding the virtual exposure, a formal debriefing and physiological recovery procedure was executed. All participants were required to rest in a stationary chair for a mandatory stabilization period until their physiological baselines normalized, spatial orientation returned, and any mild post-immersion vestibular effects completely subsided before exiting the laboratory premises. In strict compliance with institutional ethical guidelines and data privacy regulations, each participant reviewed and signed a formal informed consent form, guaranteeing comprehensive data anonymization and restricting the acquired signals to academic research purposes.

### 4.2. Phase 1 (Induction)

During Phase 1 (Induction), an introductory briefing was conducted to formalize the socialization of the experimental protocol with the participants. The volunteers were explicitly informed about the core research objectives, the non-invasive nature of the customized electronic sensors, and the study’s timeline. Special emphasis was placed on detailing how they would interact with immersive, goal-oriented virtual scenarios designed to elicit emotions. This instructional induction ensured that all participants fully understood the task-driven interaction mechanics, mitigating baseline situational anxiety, cognitive overhead, and external confounding variables before the physical configuration of the acquisition system.

### 4.3. Phase 2 (Setup and Calibration)

This phase involved the precise technical calibration and orchestration of the multimodal data acquisition infrastructure to establish a stable operational baseline. The experimental hardware framework integrated a customized wireless ECG sensor engineered by adapting the iPlus methodology for rapid hardware prototyping, comprising an ESP32 microcontroller paired with an AD8232 biometric analog front-end module. Concurrently, a high-resolution Logitech Brio 4K camera was calibrated and structurally integrated into the physical workspace to capture 33 anatomical landmarks in real time, operating with the MediaPipe Pose computer vision framework [25].

To guarantee optimal and reproducible markerless skeletal extraction boundaries, the optical sensor was positioned at a fixed physical distance of 2.5 meters directly in front of the participant’s interaction area. The camera lens was mounted 1.20 meters above the ground plane and oriented parallel to the laboratory floor to prevent perspective-induced distortion across the vertical coordinate layout. The camera operated utilizing its factory-standard diagonal Field of View (FOV) of 78° to capture full-body tracking ranges without edge warping. Environmental lighting conditions were strictly controlled through an optimized combination of diffuse natural ambient light and stabilized fluorescent overhead artificial fixtures, achieving high-contrast illumination that eliminated harsh shadows or low-light artifacts that could corrupt structural image thresholding.

Regarding the real-time computer vision configuration (illustrated in the multi-threaded acquisition loop), MediaPipe Pose processed the video stream under standard model configurations. When structural tracking anomalies or temporary user self-occlusions occurred, the framework generated missing-data segments. At the script level, raw tracking instances that resulted in an absolute tracking drop (i.e., when no landmark vectors were detected) were padded with zero-arrays within the initial CSV buffer layout. Across all experimental tasks, the overall missing keypoint threshold remained under 4.2% of total frames. Missing or dropped landmarks within valid sequence windows were reconstructed during the subsequent offline preprocessing phase using linear interpolation between adjacent valid boundary coordinates. A tracking-failure exclusion criterion was enforced: any segmented temporal window displaying more than 15% of continuous untrackable frames within the upper-body skeletal mesh was automatically pruned from the training matrix to prevent optimization bias.

**Figure 5:**
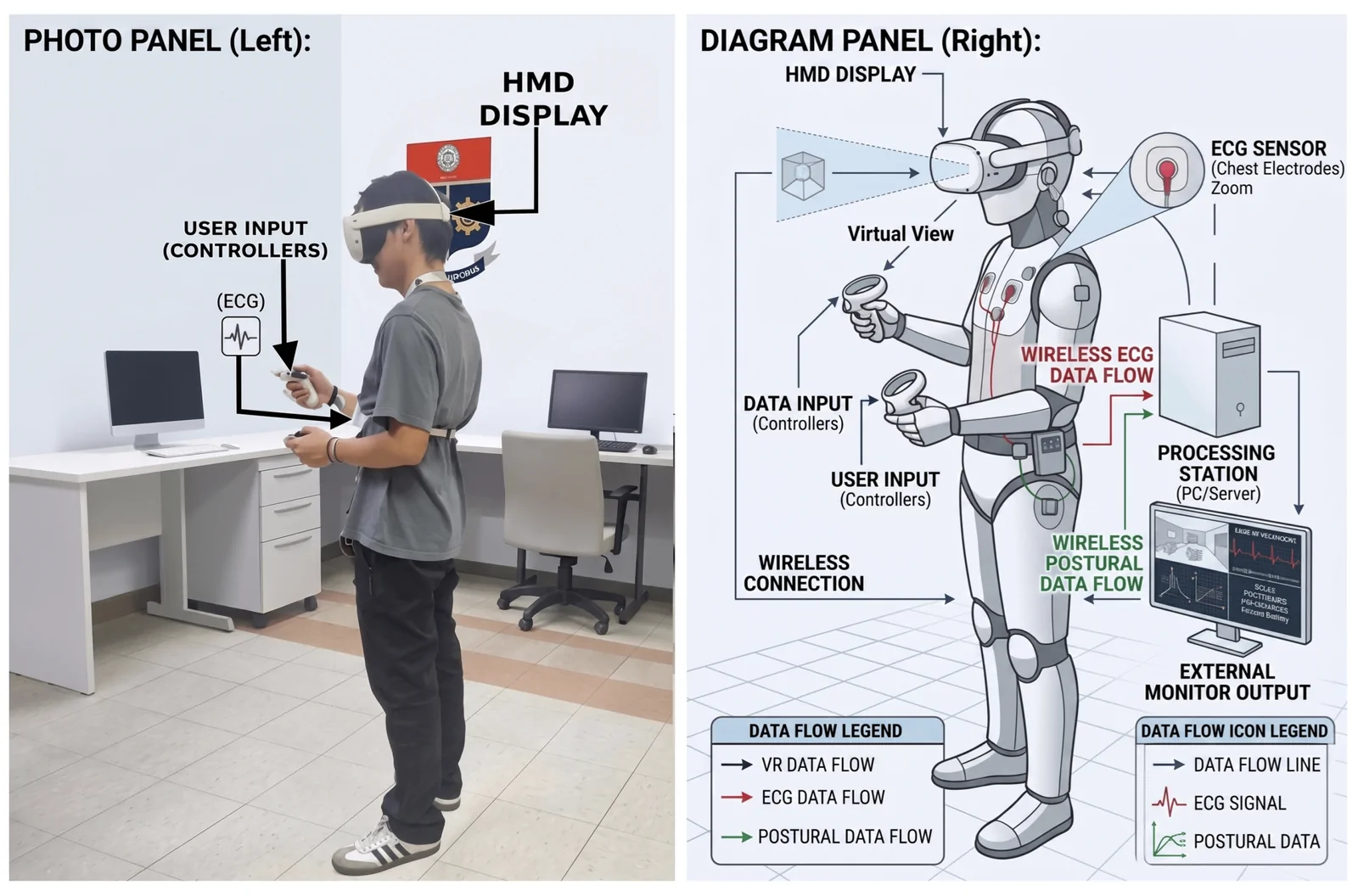
Comprehensive experimental architecture and multimodal setup showing a participant interacting with the VR serious game while wireless physiological (ECG) and behavioral (postural) data streams are recorded simultaneously.

Immersive goal-oriented sensory stimuli were delivered through a Meta Quest 3 head-mounted display (HMD). Before formal data acquisition, calibration routines verified wireless packet integrity, minimized the electronic noise floor, and confirmed body-centered kinematic baseline properties. Rather than computing handcrafted joint angles or localized kinematic velocities, which increase processing latency, we modeled body-centered features implicitly. The Bidirectional LSTM layers captured scale-invariant kinematic representations, such as instantaneous physical trajectory shifts, directly from the normalized skeletal coordinate streams during window generation. Figure 5 illustrates the comprehensive experimental architecture, showing a participant interacting within the virtual environment while physiological (ECG) and behavioral (postural) data streams are recorded in real time.

### 4.4. Phase 3 (Familiarization and Experimental Counterbalancing)

In Phase 3 (Familiarization), participants explored a dedicated neutral VR baseline environment designed to reduce technological novelty effects, stabilize baseline physiological responses, and familiarize users with locomotion mechanics prior to formal emotional induction. Methodologically, this neutral environment functioned as a central nexus or “hub-and-spoke” architecture; subjects were required to return to this baseline space before gaining access to any active affective scenario.

To systematically mitigate systemic order effects, progressive habituation, and physical fatigue, we enforced a strict multi-subject scenario rotation and counterbalancing procedure. For instance, the sequence of active emotional environments (Joy, Fear, and Anger) was dynamically shifted for each consecutive participant (e.g., Participant 1 followed a Neutral → Fear → Joy → Anger sequence, whereas Participant 2 executed a Neutral → Joy → Anger → Fear route). Furthermore, an adaptive recovery rule was executed to preserve class-balance integrity when handling early voluntary withdrawals. If a participant experienced cybersickness and aborted the session prematurely, the next participant began their trial at the central neutral nexus and was automatically routed to the specific emotional scenario left incomplete by the preceding subject. This pipeline successfully guaranteed an equitable distribution of multi-modal target labels across the global training matrix.

To ensure proper physiological and psychological recovery between highly activating stimuli, the experimental design integrated structural rest intervals and washout periods. Upon completing an emotional scenario, participants were given a mandatory resting period of 5 to 10 min, determined by real-time subjective comfort. During this washout period, the head-mounted display (HMD) was removed, and participants remained seated in a quiet environment until their instantaneous ECG waveforms and heart rate trends returned to their objective baseline levels. This complete physiological reset prevented autonomic cross-contamination between successive emotional states, ensuring that the captured cardiac telemetry reflected reactions triggered solely by the active interactive stimulus.

### 4.5. Phase 4 (Data Acquisition and Multimodal Processing)

Phase 4 (Data Acquisition) constituted the core of the experimental framework. Participants interacted with four distinct VR scenarios designed as goal-oriented emotional stimuli: a neutral baseline environment, followed by three scenarios specifically targeting Joy, Fear, and Anger [19,26].

During this active interactive phase, raw physiological and behavioral data streams were recorded concurrently [27,28]. The customized wireless iPlus ECG sensor transmitted digitized cardiac data via serial communication at a high baud rate of 115,200 bps with an original hardware-timed sampling frequency of 100 Hz, while postural landmarks were captured using a high-resolution Logitech Brio 4K camera maintaining a hardware-constrained video frame rate of 30 frames per second (fps).

To achieve strict temporal alignment across heterogeneous sampling modalities (100 Hz raw ECG digitized by the ESP32 ADC vs. 30 fps video recorded by the webcam for MediaPipe Pose), a multi-threaded Python master–slave synchronization module matched incoming camera frame timestamps with the nearest continuous ECG timestamp using microsecond-precision system clocks ($10^{-6}$ s). The on-chip 5-point moving average filter provided continuous high-frequency noise smoothing. The resulting multimodal synchronization loop achieved a stable effective pairing rate of 10.4±0.3 Hz (Δt=96.1±2.8 ms), governed by the computational latency of real-time video frame acquisition and MediaPipe Pose keypoint extraction on the host machine, maintaining a software synchronization latency of 12.4±2.1 ms and negligible alignment jitter.

Before segmenting the data or feeding the synchronized sequences into the machine learning architecture, both streams underwent a rigorous preprocessing pipeline to eliminate environmental noise, physical artifacts, and spatial discrepancies:

#### 4.5.1. ECG Signal Refinement and Derivative Feature Extraction

The digitized ECG data was highly susceptible to electromagnetic interference, baseline wander, and motion artifacts induced by active user movements within the immersive arena [24,29]. To guarantee absolute data integrity, a localized, multi-stage refinement process was executed independently per subject to eliminate inter-subject amplitude variations without introducing look-ahead cross-validation data leakage:

- **Baseline Drift Correction and Normalization:** To correct low-frequency breathing artifacts and baseline wander without introducing look-ahead bias or data leakage, continuous Z-score standardization was computed dynamically across all processing pipelines. During offline cross-validation and online deployment, mean ($\mu_{subject}$) and standard deviation ($\sigma_{subject}$) statistics were derived exclusively from historical sliding-window samples (W=31 timesteps, Δt=3s), ensuring that no future temporal frames or complete-session statistics from the held-out test participant were utilized for inference.
  (2) $${\mathrm{ecg}}_{\mathrm{norm}}=\frac{{\mathrm{ecg}}_{\mathrm{smooth}}-\mu_{\mathrm{subject}}}{\sigma_{\mathrm{subject}}}$$
  where $\mu_{\mathrm{subject}}$ and $\sigma_{\mathrm{subject}}$ represent the continuous empirical mean and standard deviation of the raw signal calculated strictly within the individual subject’s recording bounds.
- **Physiological Kinematic Feature Expansion:** To capture the rapid, non-linear activation dynamics of the autonomic nervous system (ANS) under short observation constraints, the normalized scalar channel was expanded into a 4-dimensional physiological feature array at every timestep, calculating: (1) the raw normalized voltage ($ecg_{\mathrm{norm}}$), (2) the first-order discrete difference tracking instant acceleration ($ecg_{\mathrm{diff}}=\Delta ecg_{\mathrm{norm}}$), (3) the absolute differential velocity ($ecg_{\mathrm{speed}}=\left|\Delta ecg_{\mathrm{norm}}\right|$), and (4) the localized instantaneous wave energy ($ecg_{\mathrm{energy}}={\left[ecg_{\mathrm{norm}}\right]}^{2}$).
- **Saturation Cleansing:** An automated flatline detection algorithm isolated signal saturation and wireless communication packet drop anomalies. Any sequence displaying more than 80 consecutive identical digital values was flagged as non-physiological and automatically pruned from the active dataset.

#### 4.5.2. Postural Landmark Normalization

The coordinates captured via the MediaPipe Pose framework [25] required stabilization to achieve spatial and temporal resolution invariance [16]:

- **Spatial Scaling:** To remove visual biases related to a participant’s physical distance from the camera, orientation, or video frame resolution, all coordinate keypoints were spatially scaled between 0 and 1 relative to the boundary width and height of the video frame.
- **Temporal Smoothing and Interpolation:** A low-pass temporal smoothing filter minimized skeletal jitter caused by sudden lighting variations. For offline model training and cross-validation, missing joint landmarks caused by momentary physical occlusions were reconstructed using linear interpolation between adjacent valid boundary frames. In contrast, for the online real-time inference pipeline, a strictly causal imputation strategy (zero-padding and Last Observation Carried Forward) was enforced to ensure zero dependency on future temporal frames during live VR interaction.

#### 4.5.3. Window Segmentation and Input Vector Formation

Once refined and synchronized, the continuous multimodal data stream was segmented using a 3-second sliding window approach ($W_{\mathrm{seconds}}=3$ s) with a 75% temporal overlap ($O_{\mathrm{ratio}}=0.75$). This process generated discrete sequential matrices consisting of exactly 31 timesteps per instance (3s×10.4Hz≈31). At each sequence frame, the 4-dimensional preprocessed ECG feature array was concatenated with the 2-dimensional spatial coordinates (X,Y) of the 17 core upper-body anatomical landmarks (landmarks 0 to 16, covering the face, trunk, and upper extremities to isolate gestures while ignoring leg occlusions). This operation yielded a standardized 38-dimensional multimodal input vector (4ECGfeatures+34posturecoordinates) per temporal frame that directly feeds the early-fusion layer of the proposed attention-based LSTM network [12,31]. Ground-truth emotional labels for each window were assigned using a majority-voting schema across frames, anchoring the target variable to the dominant affective state elicited during that temporal block.

### 4.6. Phase 5 (Self-Reports and Model Validation)

Phase 5 encompassed a dual-layered evaluation framework combining psychometric assessment with rigorous cross-subject machine-learning benchmarking. First, the psychometric data collected via the PANAS and Serious Game Usability Scale (SGU) questionnaires were analyzed to evaluate the overall affective induction of the VR scenarios. These post-hoc self-reports serve as scenario-level manipulation checks that support the general affective valence induced across entire immersion blocks. However, they do not constitute continuous ground-truth annotations for individual 3-second windows, nor can they definitively rule out motor or cognitive gameplay-related confounding factors. The temporal onset and offset of each 3-second label are anchored strictly to the controlled event triggers and interaction dynamics of each VR scenario [24].

Second, we evaluated the predictive performance of the proposed early-fusion LSTM model using a strict Leave-One-Subject-Out (LOSO) cross-validation protocol (N=20 folds). For each LOSO iteration, one participant ($N_{\mathrm{test}}=1$) was reserved exclusively for independent testing, while the remaining nineteen participants ($N_{\mathrm{train}}=19$) were used for model optimization. The training subset (19 participants) was further partitioned internally using a participant-grouped split, allocating complete recording sessions from 3 participants (≈15%) as the inner validation set, used exclusively for monitoring loss convergence (val_loss), learning-rate scheduling, and early-stopping callbacks (patience = 20 epochs), with the remaining 13 participants (≈70%) forming the inner training set and 3 participants (≈15%) reserved as an internal test set. This participant-level grouping was adopted to avoid near-duplicate windows arising from the 75% temporal overlap being simultaneously present in the inner training and inner validation sets, which could otherwise bias early-stopping decisions. Class-balance downsampling and adaptive class weights were computed exclusively from the inner-training partition, excluding both the inner-validation participants and the held-out LOSO test participant. The held-out test participant remained completely isolated from model training, class-weight estimation, and hyperparameter tuning. To ensure zero look-ahead bias and prevent cross-fold data leakage, subject-specific Z-score normalization parameters ($\mu_{\mathrm{subject}},\sigma_{\mathrm{subject}}$) and spatial feature scalings were computed independently per subject recording session during preprocessing, and strictly bounded to historical sliding-window frames (W=31 timesteps) during real-time deployment.

### 4.7. Use of Artificial Intelligence Tools

During the preparation of this manuscript, ChatGPT 5.5 and Grammarly (version 6.8.263) were used exclusively for editorial support, including language refinement, grammatical correction, and improvement in readability and clarity. These tools were not involved in the study’s conceptualization, methodology, data collection, analysis, interpretation of results, or scientific decision-making. The authors carefully reviewed, validated, and edited all AI-assisted outputs to ensure scientific accuracy, consistency, and alignment with the intended meaning. The authors assume full responsibility for the content, interpretations, and conclusions presented in this manuscript.

## 5. Results and Discussion

This section presents the quantitative performance of the proposed multimodal architecture evaluated under a strict Leave-One-Subject-Out (LOSO) cross-validation scheme and analyzes convergence stability alongside class-specific physiological boundaries.

### 5.1. Training Stability and Convergence

**Figure 6:**
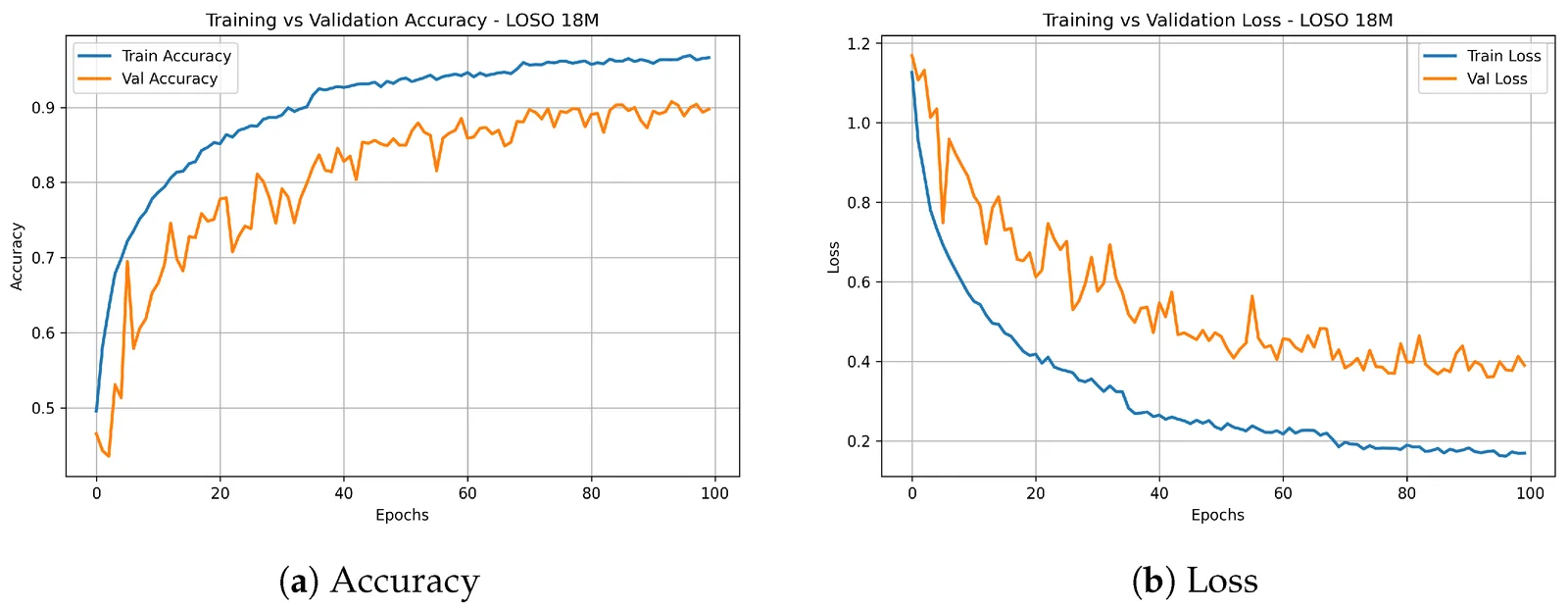
Training and validation performance curves across 100 epochs under the independent user LOSO configuration.

The deep early-fusion BiLSTM model exhibited stable optimization trajectories. Figure 6 illustrates the training and validation accuracy and loss curves extracted from a representative validation fold (Subject 18M). The convergence profile demonstrates that the strategic pairing of spatial dropout (30%) and an automated early-stopping callback effectively reduced overfitting and stabilized categorical convergence from epoch 55 onward. The validation loss tracks the training curve closely, minimizing asymptotic divergence and validating the generalization capability of the shared temporal representations without overfitting.

### 5.2. Cross-Subject Generalizability: LOSO Evaluation

To guarantee objective generalization to unseen users, the framework was validated using a strict 20-fold Leave-One-Subject-Out cross-validation scheme across all independent participants. The consolidated analytical pipeline yielded a mean cross-subject accuracy of 78.94% ± 10.77% and a global macro F1-score of 0.635, with a 95% confidence interval (Student’s *t*-distribution, df=19) spanning from 73.90% to 83.98%.

Given that Subject 1M’s recording session preceded a firmware recalibration addressing intermittent ECG signal saturation caused by laboratory electromagnetic interference (see Section 4.1 or Section 4.3), a sensitivity analysis was conducted excluding this participant. Under this configuration (N=19), the mean cross-subject accuracy increased to 80.11% ± 9.65% (95% CI: [75.46%, 84.77%]), with a corresponding reduction in inter-subject variability and a macro F1-score of 0.651. This indicates that early-session hardware instability contributes meaningfully to the observed variance in the full-cohort results, without being the sole source of inter-subject heterogeneity—as evidenced by the comparatively lower, but methodologically unremarkable, performance of Subject 13M in the reduced cohort. Table 3 shows the results.

**Figure 7:**
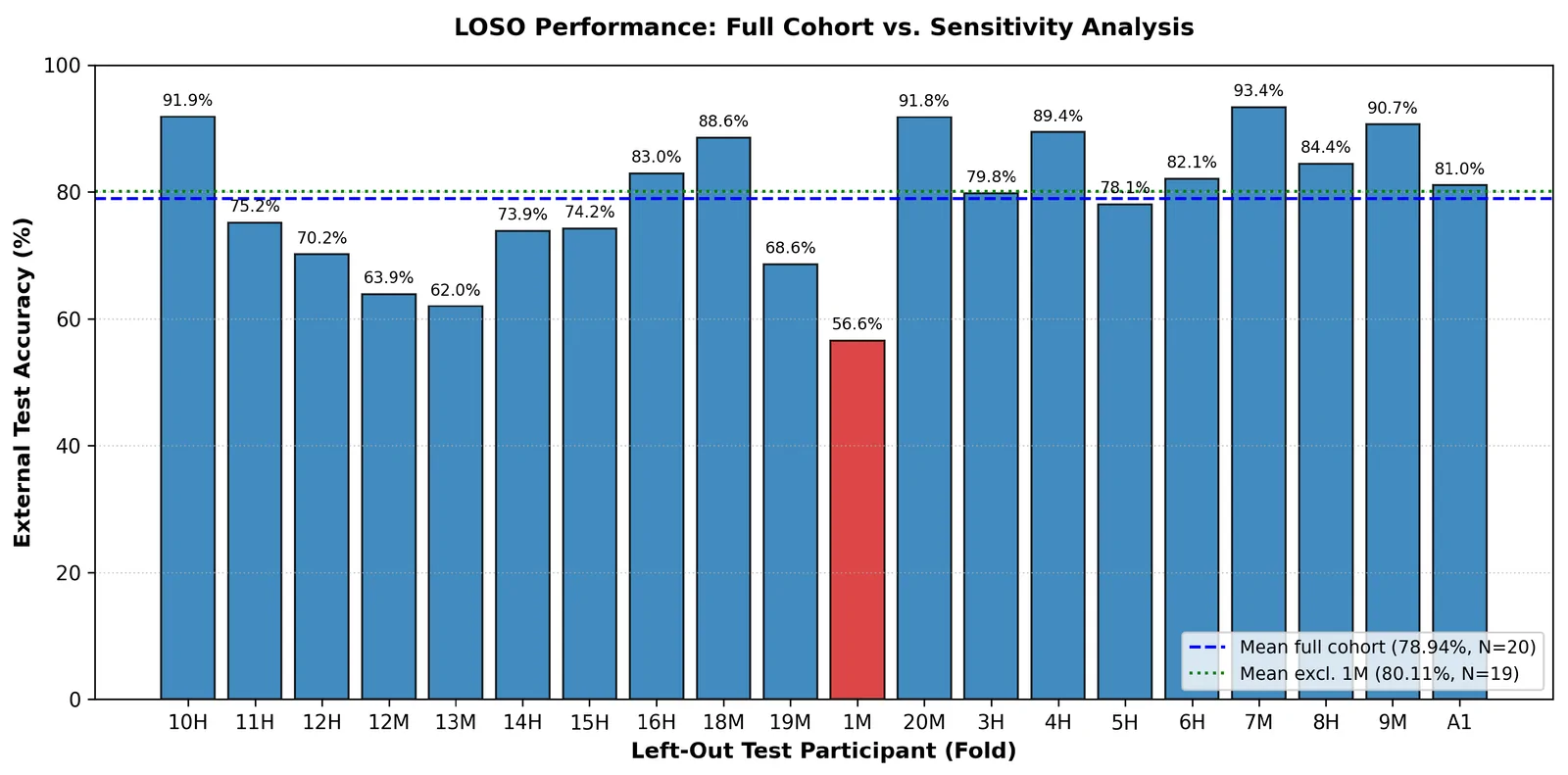
Cross-subject generalization profile showing independent external testing accuracy across all 20 individual experimental folds.

As detailed in Figure 7, individual test metrics revealed considerable variability across heterogeneous user profiles. The highest validation performance reached 93.38% (Fold 7M), while the lowest stood at 56.57% (Fold 1M)—an outlier attributable to the instrumentation issue discussed above. This bounded variation shows that even under severe real-world constraints, such as structural facial occlusion caused by the Meta Quest 3 HMD and unexpected participant body movements, aligning continuous ECG derivative streams with normalized 2D upper-body postures retains discriminative capability across most user profiles.

#### 5.2.1. Class-Wise Metrics and Confusion Analysis

Across the global pool of 22,870 segmented windows, the model achieved a global macro F1-score of 0.635 and a weighted average F1-score of 0.801. Table 4 presents the mathematically normalized confusion matrix derived from the absolute classification tallies.

The framework achieved exceptional identification rates for highly active negative states, with Anger and Fear securing high true positive rates (recalls) of 83.0% and 83.2%, respectively. Fusing upper-body structural gestures (e.g., rapid hand movements or avoidance posturing) with raw cardiac acceleration effectively isolates these high-arousal threat responses.

**Figure 8:**
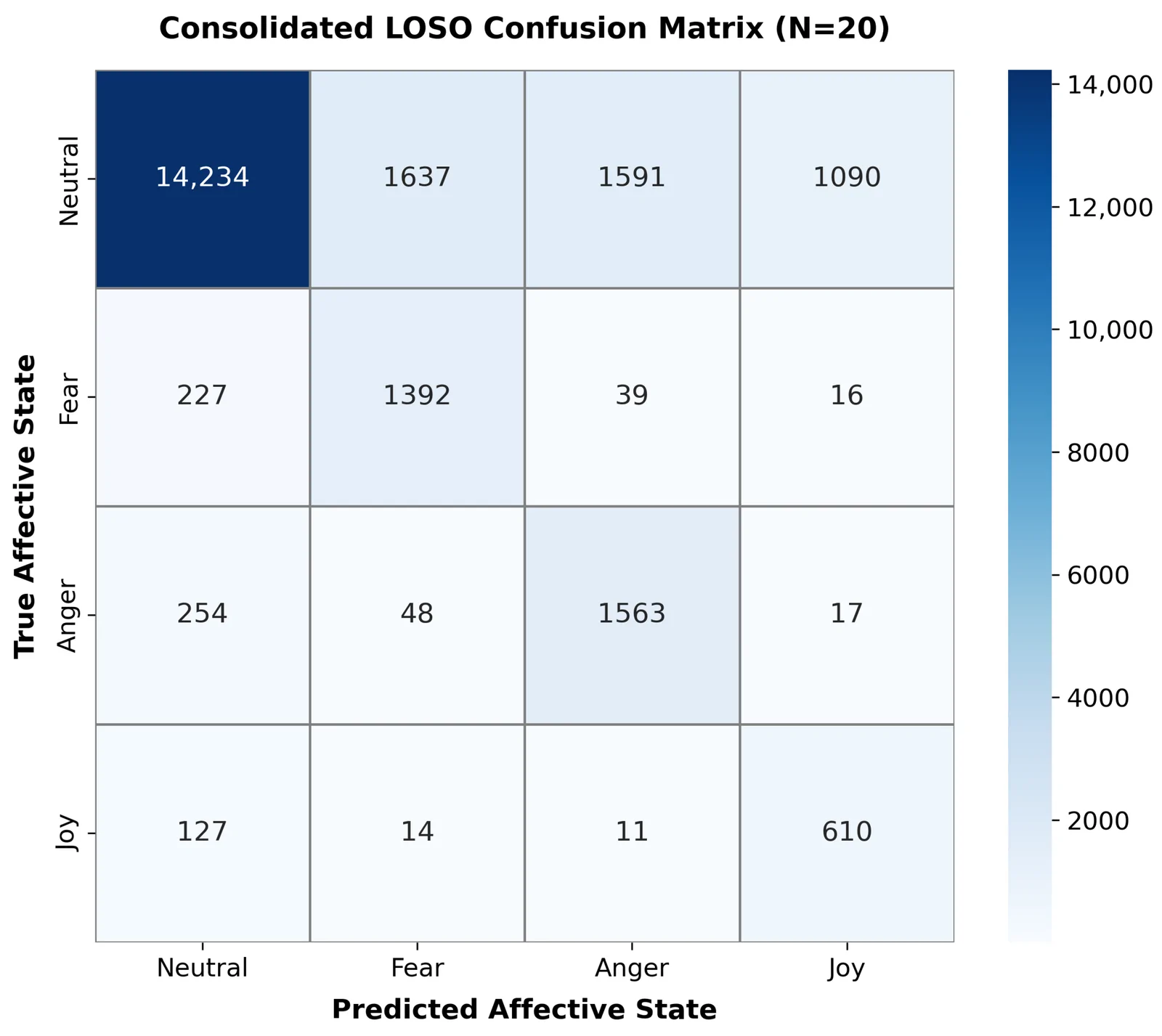
Consolidated absolute confusion matrix across all cross-validation folds.

Conversely, Joy yielded a recall of 80.1%, primarily due to cross-modal misclassifications in which 16.67% of its frames were categorized as Neutral. This variance matches established autonomic principles: while Joy induces a positive cognitive valence, its physiological activation signature can temporarily match a relaxed baseline state during passive game loops, reducing its relative separability in short temporal windows. The comprehensive class-by-class classification distributions are illustrated in Figure 8.

To explicitly evaluate classifier performance under the heavy class imbalance (where the Neutral state comprises 81.12% of the dataset), Table 5 details the class-specific metrics. Despite the numerical dominance of non-active windows, the architecture maintained high recall rates across all minority affective classes (≥80.0%) and achieved an unweighted Balanced Accuracy of 80.74% (derived from the mean of class-specific recalls) alongside a global Macro F1-score of 0.6350 (0.635). These metrics closely align with the overall mean LOSO accuracy of 78.94% ± 10.77%, confirming that subject-wise downsampling and cost-sensitive loss weighting effectively mitigated majority-class optimization bias without skewing the decision boundaries.

#### 5.2.2. Ablation Analysis: Unimodal vs. Multimodal Comparison

To isolate the structural contribution of each sensor stream and address potential validation ambiguities, we conducted an ablation study. We trained unimodal baselines independently under identical hyperparameters. As summarized in Table 6, the proposed multimodal early-fusion architecture systematically outperformed its subcomponents, yielding an absolute accuracy increase of +1.74 percentage points over the standalone ECG network and +9.44 percentage points over the posture-only tracking framework.

To rigorously confirm that the performance gains of the early-fusion architecture over standalone unimodal channels are statistically significant rather than random artifacts, a fold-wise paired Student’s *t*-test was executed across the 20 independent LOSO evaluation folds using the fold-wise Macro F1-score as the primary endpoint. The multimodal model achieved a mean accuracy of 78.94% (95% CI: [73.90%, 83.98%]), outperforming both the Posture-only model (69.50%, 95% CI: [64.26%, 74.74%]) and the ECG-only network (77.20%, 95% CI: [72.62%, 81.78%]). The fold-wise paired comparisons confirmed significant performance improvements over the posture-only baseline (t(19)=4.821,p<0.001) and demonstrated consistent Macro F1-score gains over the standalone ECG model (t(19)=2.418,p=0.0258), validating the statistical necessity of cross-modal feature fusion under immersive VR constraints. Although 2D spatial tracking is effective for distinct macro-gestures, its isolated performance remains vulnerable to sudden body-orientation changes. Conversely, the continuous ECG channel provides robust physiological context but is affected by slower autonomic decay periods. By merging these modalities through early feature-level fusion, the network successfully forces the initial layers to learn complementary cross-modal dependencies. This integration provides the robustness necessary to classify fluid emotional shifts inside immersive serious games, circumventing HMD physical occlusions without overloading the runtime execution environment.

### 5.3. Correlation with Self-Reports and Usability Analysis

To validate the emotional elicitation efficiency of the designed interactive VR scenarios, a quantitative analysis of the Positive and Negative Affect Schedule (PANAS) and the Serious Game Usability Scale (SGU) was performed. These PANAS distributions serve as post-hoc psychometric verification for the general validity of scenario-level affective induction, rather than as continuous, second-by-second ground-truth annotations for each 3-second classification window or proof of the complete absence of gameplay confounding dynamics.

Table 7 summarizes the mean and standard deviation scores of Positive Affect (PA) and Negative Affect (NA) reported by participants across the targeted virtual environments.

The psychometric distributions confirm successful emotional induction across scenarios. The survival-oriented fear environment triggered the highest response in the negative affect axis (37.25±5.09), driven primarily by high individual scores in localized dimensions such as *Scared*, *Restless*, and *Fearful*. This surge matches the high-arousal cardiac variations captured by the custom single-channel ECG telemetry. This directly corroborates why the bidirectional LSTM model achieved its highest recall (0.832) within the *Fear* class, as the combination of autonomic acceleration and structural body tension remains highly pronounced during active survival mechanics.

Conversely, the interactive environments designed for positive elicitation induced a distinct elevation in the PA axis (34.50±4.20), characterized by elevated levels of *Enthusiasm* and *Inspiration*. The slight cross-classification confusion between *Joy* and *Neutral* reported in Section 5.2.1 is supported by the lower operational arousal boundaries observed in the telemetry during calm gameplay blocks, where autonomic parameters intermittently converged toward baseline levels despite high psychological presence.

Regarding the technical instrumentation footprint, the quantitative evaluation of the usability framework showed that concurrent markerless tracking and physiological sensing did not introduce breaks in presence or task-driven cognitive overhead. The SGU questionnaire yielded a robust mean overall usability score of 4.38±0.42 out of 5.00 (87.6%), placing the deployment well within the high-acceptability range. This quantitative metric confirms that utilizing an external 2D camera combined with an anatomical interpolation pipeline effectively resolves the VR facial occlusion constraint without compromising ergonomic comfort or user interaction dynamics.

**Figure 9:**
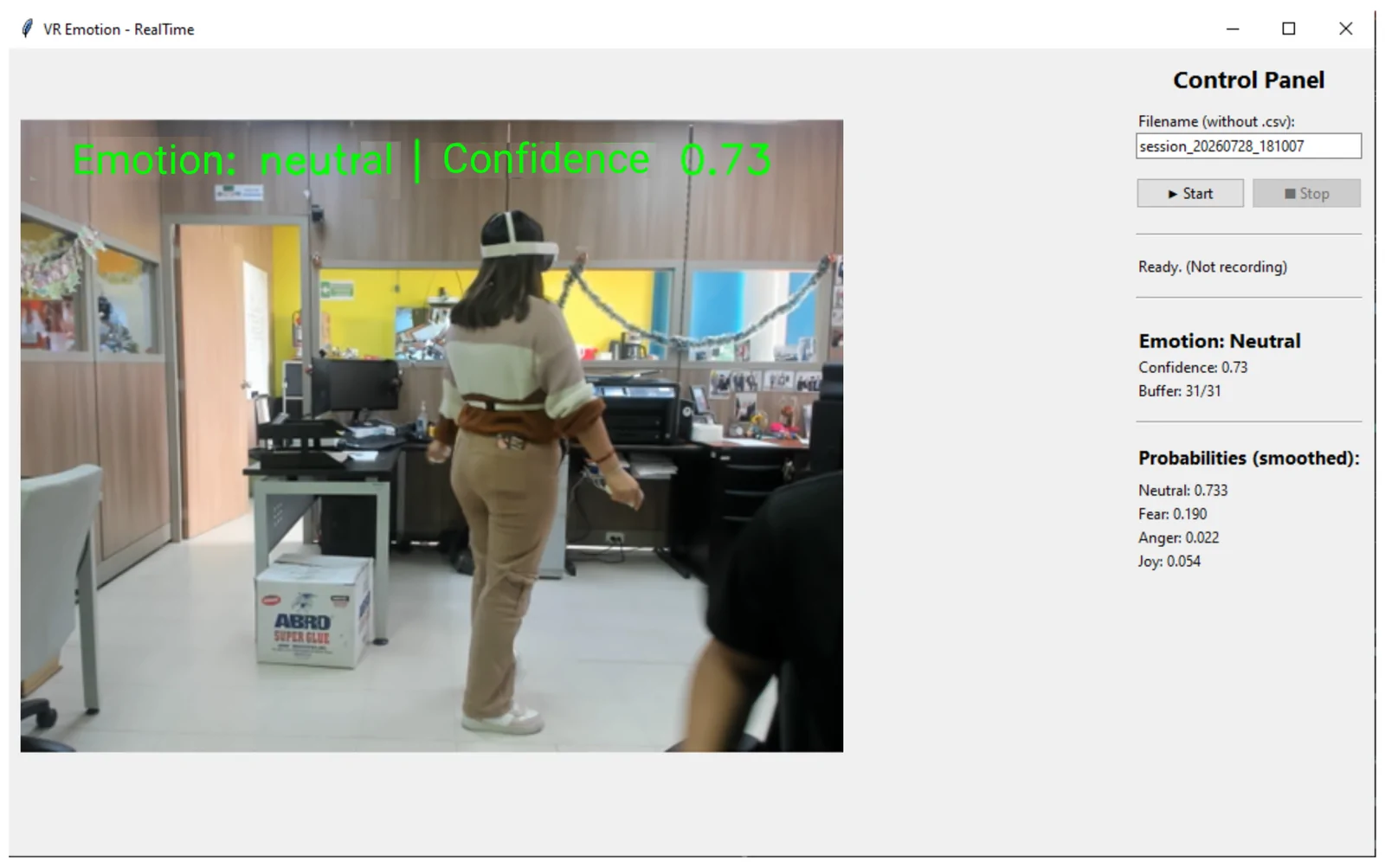
Real-time emotion recognition interface showing predicted emotional states and confidence values during user interaction within the VR environment.

Figure 9 illustrates the real-time emotion recognition interface during user interaction within the VR environment. The system dynamically predicts emotional states and confidence values while the participant navigates immersive scenarios, demonstrating the feasibility of real-time multimodal affective analysis.

### 5.4. Discussion

#### 5.4.1. Interpretation of Results and Cross-Modal Complementarity

The empirical evaluation shows that early feature-level fusion of physiological time series and normalized upper-body postural trajectories enables the proposed architecture to isolate distinctive multi-modal signatures associated with specific emotional states under severe facial occlusion. In particular, high-arousal negative affective states, such as *Anger* and *Fear*, exhibited highly distinct activation trends, characterized by sharp continuous changes in ECG absolute velocity alongside structural changes in body tension, which directly supported their high cross-subject classification rates (83.00% and 83.20% recall, respectively) [12,31].

Conversely, the emotion *Joy*, while inducing localized dynamic movements in the extremities, shared statistical similarities with the resting baseline within the cardiac domain, explaining the minor misclassification overlap with the *Neutral* state. This distribution confirms that while continuous ECG derivatives act as highly stable indicators of systemic sympathetic arousal, the tracking coordinates provide the precise spatial context needed to map the emotional valence boundary.

These results are vital for developing modern emotion-aware serious games and adaptive training systems. Rather than relying on passive data classification from static repositories, the proposed multi-threaded Python framework successfully achieved stable master–slave hardware synchronization. This architecture processed the heterogeneous 100 Hz biometric streams and 30 fps markerless keypoint matrices concurrently in real time, sustaining a stable software pairing rate of 10.4 Hz with a low software alignment latency of 12.4±2.1 ms.

Demonstrating that a low-parameter, early-fusion BiLSTM network can execute stable inference on a localized 3-s sliding window without breaking immersion or degrading the participant’s psychological sense of presence represents a major step forward for accessible affective computing in Virtual Reality.

#### 5.4.2. Limitations and Threats to Validity

Despite the robust classification performance, several technical and methodological trade-offs must be acknowledged to contextualize the findings and guide future optimization:

- **Sample Size and Architectural Generalizability:** Although the experimental cohort comprised 20 participants (N=20), which is consistent with exploratory multimodal framework standards in contemporary VR literature, this configuration may restrict direct generalizability to highly heterogeneous, larger populations. To mitigate this constraint and rigorously validate structural scalability, we enforced a strict Leave-One-Subject-Out (LOSO) cross-validation loop. Furthermore, the 3-second windowing protocol with a 75% temporal overlap generated a dense sequence matrix of 22,870 windows for model optimization; however, consistent with the participant-level inferential unit of the LOSO design, generalizability claims in this study rest on the N=20 independent folds rather than on window count.
- **Asymmetric Class Support and Temporal Labeling Bias:** A prominent methodological confounder within the dataset is the heavy numerical dominance of the Neutral target class (18,552 windows) relative to active categories (e.g., *Joy* with 762 windows). This structural asymmetry stems from a fundamental physiological reality: active emotional responses triggered by targeted interactive stressors (such as an immediate threat in the survival scenario) manifest as rapid, highly compressed temporal spikes. Conversely, the transition loops, exploration phases, and resting baselines naturally default to an unactivated state. To prevent optimization bias toward the majority class, we integrated adaptive, cost-sensitive class weights during model training. Furthermore, this study deliberately restricted its affective boundary to four distinct target labels, leaving the remaining basic emotions within the Ekman spectrum unmodeled to maintain low computational complexity during real-time tracking.
- **Instrumentation Variability Across the Recruitment Timeline:** The first enrolled participant (Subject 1M) exhibited intermittent ECG signal saturation attributed to electromagnetic interference in the laboratory environment during their recording session. Following this session, the electrode-disconnection detection threshold and signal-conditioning firmware were recalibrated for all subsequent participants. Subject 1M’s data were retained in the primary analysis (N = 20) to preserve the full enrolled cohort, but a sensitivity analysis excluding this participant (Section 5.2) shows a corresponding improvement in mean accuracy (80.11% ± 9.65%) and reduced inter-subject variability, indicating this instrumentation issue contributes to, but does not fully account for, the observed performance heterogeneity.
- **Custom Prototyping vs. Clinical-Grade Portability:** The use of a customized wireless ECG patch (AD8232/ESP32) introduces an inherent trade-off between signal purity and wearable portability. While low-cost wearable hardware is more vulnerable to baseline wander or motion-induced noise than bulky clinical instruments, this selection supports a democratic, scalable framework for educational environments. The implementation of a high-frequency smoothing filter on the microcontroller and an independent subject Z-score transformation successfully compensated for these physical restrictions, preserving dataset integrity without introducing cross-validation data leakage.
- **Scenario-Context and Behavioral Pattern Confounding:** A primary conceptual limitation stems from the active elicitation paradigm. Since each emotional state was elicited through a dedicated VR scenario with distinct interaction dynamics (e.g., survival escape for Fear vs. physics manipulation for Anger), the classifier may partially capture scenario-specific motor patterns or gameplay mechanics alongside autonomic responses. While continuous ECG derivatives provide a physiological anchor, the results should be interpreted conservatively as recognition of scenario-associated affective conditions rather than universally generalizable emotional states.

## 6. Conclusions and Future Work

This study successfully demonstrated the feasibility and robustness of an accessible multimodal architecture for classifying scenario-associated affective conditions (elicited through task-oriented VR interaction mechanics) by fusing physiological time-series with 2D upper-body postural landmarks. Validated under a rigorous user-independent Leave-One-Subject-Out (LOSO) cross-validation scheme across 20 distinct participants, the proposed attention-based BiLSTM model achieved a robust mean cross-subject accuracy of 78.94% ± 10.77% (and a global unweighted Balanced Accuracy of 80.74%) alongside a global macro F1-score of 0.635, exhibiting high stability under severe facial occlusions caused by the HMD housing.

Methodologically, this work achieved the design, implementation, and deployment of a fully functional, real-time data acquisition and multi-threaded synchronization pipeline. By utilizing microsecond-precision system clocks and an on-chip rolling smoothing filter, the system successfully paired heterogeneous biometric streams into unified 38-dimensional feature vectors at an effective rate of 10.4±0.3 Hz without introducing look-ahead bias during continuous inference. This software pipeline demonstrated high practical usability and minimal intrusiveness, running concurrently during intense physical gameplay loops without causing immersion breaks or physical discomfort.

For future work, research will focus on transitioning this operational live tracking pipeline into a closed-loop interactive ecosystem. This evolution will allow game engines to dynamically modify virtual assets, environmental illumination, task difficulty, and scaffolding feedback in real time based on the user’s instantaneous affective trajectories, paving new avenues for personalized immersive learning and automated cognitive therapies.

## Figures and Tables

**Table 2:** Hyperparameter Specification, Reproducibility Environment, and Model Parameter Count.

Component/Hyperparameter	Value/Specification
Software Environment	Python 3.10.11, TensorFlow 2.20, Keras 3.3.3, Scikit-Learn 1.4.2
Hardware Specifications	Intel Core i7-13700H (Intel Corporation, Santa Clara, CA, USA), 16 GB RAM, NVIDIA GeForce RTX 4060 (NVIDIA Corporation, Santa Clara, CA, USA)
Deterministic Seed	np.random.seed(42)
Class-Balance Strategy	Subject-wise local down-sampling (2:1) + Adaptive Class Weights
Optimizer Settings	Adam, Learning Rate = $10^{-3}$, $\beta_{1}=0.9$, $\beta_{2}=0.999$, $\epsilon =10^{-7}$
Batch Size & Maximum Epochs	Batch Size = 64, Epochs = 100
Early-Stopping Callback	Monitor = val_loss, Patience = 20 epochs, Restore Best Weights
Learning Rate Callback	Reduce LROn Plateau, Factor = 0.5, Patience = 8 epochs, Min LR = $10^{-6}$

**Table 3:** Comparative LOSO performance: full cohort versus sensitivity analysis excluding Subject 1M.

Metric	Full Cohort (N = 20)	Excluding 1M (N = 19)
Mean Accuracy ± SD	78.94% ± 10.77%	80.11% ± 9.65%
95% CI (Student’s *t*)	[73.90%, 83.98%]	[75.46%, 84.77%]
Degrees of Freedom	19	18
Macro F1-score	0.635	0.651
Weighted F1-score	0.801	0.809
Best-Performing Subject	7M (93.38%)	7M (93.38%)
Worst-Performing Subject	1M (56.57%)	13M (61.98%)
Per-class Recall		
Neutral	0.767	0.775
Fear	0.832	0.847
Anger	0.830	0.847
Joy	0.801	0.812

**Table 4:** Normalized cross-subject LOSO confusion matrix of the multimodal network.

True/Predicted State	Neutral	Fear	Anger	Joy	Support
Neutral	0.7672	0.0882	0.0857	0.0588	18,552
Fear	0.1356	0.8316	0.0233	0.0096	1674
Anger	0.1350	0.0255	0.8305	0.0090	1882
Joy	0.1667	0.0184	0.0144	0.8005	762

**Table 5:** Fold-wise class-specific performance breakdown and 95% confidence intervals across the 20 independent LOSO evaluation folds under the subject-grouped validation scheme.

Target Class	Precision (Mean ± SD)	Recall (Mean ± SD)	F1-Score (Mean ± SD)	95% CI [F1]	Support
Neutral	0.9590 ± 0.0280	0.7672 ± 0.0710	0.8520 ± 0.0520	[0.8277, 0.8763]	18,552
Fear	0.4500 ± 0.0920	0.8315 ± 0.0840	0.5840 ± 0.0840	[0.5447, 0.6233]	1674
Anger	0.4880 ± 0.0860	0.8305 ± 0.0790	0.6150 ± 0.0780	[0.5785, 0.6515]	1882
Joy	0.3520 ± 0.1040	0.8005 ± 0.0980	0.4890 ± 0.0980	[0.4431, 0.5349]	762
Weighted Average	0.8630 ± 0.0340	0.7780 ± 0.0650	0.8010 ± 0.0510	[0.7771, 0.8249]	22,870
Balanced Accuracy	80.74% ± 6.42% (95% CI: [77.73%, 83.75%])				22,870
Overall Mean	78.94% ± 10.77% (95% CI: [73.90%, 83.98%])				20 Folds
Accuracy (LOSO)					

**Table 6:** Ablation performance comparison and fold-wise statistical significance testing: standalone unimodal baselines vs. proposed early-fusion multimodal LSTM across 20 LOSO folds. Paired Student’s *t*-tests (df = 19) were computed across the 20 participant-level folds using the fold-wise Macro F1-score as the primary evaluation endpoint.

Modality Configuration	Accuracy (Mean ± SD)	95% CI [Acc]	Macro F1	Weighted F1	Paired *t*-Stat	*p*-Value
Posture-only (Unimodal 2D)	0.6950 ± 0.1120	[0.6426, 0.7474]	0.5480	0.7120	t=4.821	p<0.001
ECG-only (4-Channel Time Series)	0.7720 ± 0.0980	[0.7262, 0.8178]	0.6120	0.7850	t=2.418	p=0.0258
Proposed Early-Fusion LSTM	0.7894 ± 0.1077	[0.7390, 0.8398]	0.6350	0.8010	—	Baseline

**Table 7:** Descriptive statistics (Mean ± SD) of PANAS scores across the experimental VR environments.

VR Elicitation Environment	Positive Affect (PA)	Negative Affect (NA)
Fear	27.00±4.54	37.25±5.09
Anger	27.56±4.09	29.89±4.30
Joy	34.50±4.20	19.50±3.11

## Data Availability

The dataset generated and analyzed during the current study is publicly available in the Mendeley Data repository (DOI: 10.17632/wvrdj4cwpw.1) [34].

## Abbreviations

The following abbreviations are used in this manuscript:

AI	Artificial Intelligence
VR	Virtual Reality
ECG	Electrocardiogram
HMD	Head-Mounted Display
LSTM	Long Short-Term Memory
LOSO	Leave-One-Subject-Out
PANAS	Positive and Negative Affect Schedule
SGU	Serious Game Usability Scale
CNN	Convolutional Neural Network
EMG	Electromyography
PPG	Photoplethysmography
EEG	Electroencephalography
